# Cloning, molecular and functional characterization by overexpression in *Arabidopsis* of MAPKK genes from grapevine (*Vitis vinifera*)

**DOI:** 10.1186/s12870-020-02378-4

**Published:** 2020-05-07

**Authors:** Gang Wang, Ying-hai Liang, Ji-yu Zhang, Zong-Ming ( Max) Cheng

**Affiliations:** 1 College of Horticulture, Nanjing Agricultural University, Nanjing, 210095 Jiangsu China; 2 Institute of Botany, Jiangsu Province and Chinese Academy of Sciences, Nanjing, 210014 Jiangsu China; 3 Institute of Pomology, Jilin Academy of Agricultural Sciences, Gong Zhuling, Jilin Province, 136100 China; 4 Department of Plant Sciences, University of Tennessee, Knoxville, Tennessee 37996 USA

**Keywords:** Grapevine, Mitogen-activated protein kinase kinase (MAPKK/MKK), Expression analysis, Salt and drought stresses, Transgenic plants

## Abstract

**Background:**

The mitogen-activated protein kinases (MAPKs), as a part of the MAPKKK-MAPKK-MAPK cascade, play crucial roles in plant development as an intracellular signal transduction pathway to respond various environmental signals. However, few MAPKK have been functionally characterized in grapevine.

**Results:**

In the study, five MAPKK (MKK) members were identified in grapevine (cultivar ‘Pinot Noir’), cloned and designated as VvMKK1-VvMKK5. A phylogenetic analysis grouped them into four sub-families based on the similarity of their conserved motifs and gene structure to *Arabidopsis* MAPKK members. qRT-PCR results indicated that the expression of VvMKK1, VvMKK2, VvMKK4, and VvMKK5 were up-regulated in mature leaf and young blades, and roots, but exhibited low expression in leaf petioles. VvMKK2, VvMKK3, and VvMKK5 genes were differentially up-regulated when grapevine leaves were inoculated with spores of *Erisyphe necator*, or treated with salicylic acid (SA), ethylene (ETH), H_2_O_2,_ or exposed to drought, indicating that these genes may be involved in a variety of signaling pathways. Over expression of VvMKK2 and VvMKK4 genes in transgenic *Arabidopsis* plants resulted in the production of seeds with a significantly higher germination and survival rate, and better seedling growth under stress conditions than wild-type plants. Overexpression of VvMKK2 in *Arabidopsis* improved salt and drought stress tolerance while overexpression of VvMKK4 only improved salt stress tolerance.

**Conclusions:**

Results of the present investigation provide a better understanding of the interaction and function of MAPKKK-MAPKK-MAPK genes at the transcriptional level in grapevine and led to the identification of candidate genes for drought and salt stress in grapes.

## Background

Plants, being sessile organisms, have developed signaling mechanisms to regulate their cellular metabolism and allows them to adapt to environmental changes and stress stimuli. In fact, plants have evolved diverse signaling networks that function in sensing stress and transmitting stress signals that initiate changes in gene expression [1]. Mitogen-activated protein kinase (MAPK) signaling cascades function as a common signal transduction module that translates external stimuli into a cellular response, and is involved in a variety of biological processes [2]. The classical MAPK signaling cascade is composed of a linear cascade of three specific classes of serine/threonine protein kinases: MAPK, MAPK kinase (MAPKK/MAP 2 K/MKK), and MAPK kinase kinase (MAPKKK/MAP 3 K/MEKK). Quite a few MAPKKK-MAPKK-MAPK genes have been characterized in *Arabidopsis* and other plants [3]. They all function as upstream and downstream regulators via phosphorylation [3, 4]. MAPKs are phosphorylated at threonine and tyrosine residues in the T-X-Y motif and are activated by their specific MAPKKs, which their self are phosphorylated at two serine/threonine residues located within the S/T-XXXXX-S/T motif and are activated by upstream MAPKKKs [5–8]. Upon activation, the MAPK could be regulate the expression of downstream genes through phosphorylation of transcription factors or components of transcription machinery [4, 9]. Numerous studies have provided evidence that MAPK cascades play an important role in the transduction of diverse cellular processes when plant cells are exposed to a variety of abiotic and biotic stresses, including drought, salinity, and temperature (both high and low) stress, as well as pathogen attack, and plant hormone response [2, 10–14]. MAPK cascade is also responsible for regulating seed dormancy in cereal such as barley [15].

Many members of MAPK cascades have been identified in a variety of plant species as the number of whole genome sequences have increased and subjected to analysis. MAPKK gene family is one of the most important components of MAPK cascades. For example: the *Arabidopsis thaliana* genome contains 80 MAPKKKs, 10 MAPKKs, and 20 MAPKs [11]; rice contains 75 MAPKKKs, 8 MAPKKs, and 17 MAPKs [16, 17]; tomato contains 89 MAPKKKs, 6 MAPKKs, and 16 MAPKs [18, 19]. The number of MAPKKs in these species are close to half of the number of MAPKs and much lower than the number of MAPKKKs. These data suggest MAPKKs play an important role in integrating signals from several MAPKKKs and in transfusing signals to various MAPKs. MAPKKs may function as bifurcation points and are likely to be involved in multiple MAPK cascades that are activated in response to a variety of stresses [1, 11]. Several MAPKKs have been identified in different plant species, including *Arabidopsis* MKK1 and MKK2–5, tobacco NtMEK1–2, alfalfa SIMKK, tomato LeMEK1, and maize ZmMEK1 and ZmMKK3–4 [20]. The *Arabidopsis* MKK1/MKK2-MPK4/MPK6 cascades have been previously demonstrated to play an integral role in plant response to salt and cold stress, as well as pathogen attack [21–25]. *Arabidopsis* MKK3 participates in signaling cascades induced by pathogen infection [26]. *Arabidopsis* MKK4/MKK5-MPK3/MPK6 cascades play an important role in the regulation of biotic stress [27, 28]. AtMKK6 directly regulates cytokinesis and mitosis [29]. NtMEK2 activates SA-induced protein kinase (SIPK) and wound-induced protein kinase (WIPK) in tobacco, which induce cell death [30]. SIMKK mediates both salt and elicitor-induced signals in alfalfa [31]. ZmMKK4 confers salt and cold tolerance in *Arabidopsis*, while ZmMKK4 and ZmMKK3 positively mediate osmotic stress in transgenic tobacco by scavenging reactive oxygen species [32].

Grapevine (*Vitis vinifera* L.) is one of the valuable and widely-grown fruit crops in the world. Although MAPK cascades are involved in transducing multiple defense and stress signals, the role of MAPK cascades in grapevine in response to biotic and abiotic stresses has not been elucidated. In our previous studies, MAPK and MAPKKK gene families in grapevine were identified and their expression in different organs and under different stress conditions was analyzed [7, 33]. More recently, using the *V. vinifera* genome (http://genomes.cribi.unipd.it/grape/) [34], members of the MAPKK gene family in grapevine were identified and subjected to a phylogenetic analysis, however, no experimental evidence on their expression and function was provided [3]. In addition, expression or functional analyses of MAPKK gene family members have not been reported for their role in biotic and abiotic stress response. In this study, five MAPKK genes were identified in the grapevine genome through homology searches and all five were cloned by polymerase chain reaction (PCR). Their phylogenetic relationships, conserved motifs, and gene structure were also compared to known MAPKK genes in *Arabidopsis.* Subsequently, the expression level of each of the MAPKK genes was analyzed in different grapevine tissues and in response to different hormones and biotic and abiotic stresses. Lastly, two MAPKK genes were identified that may be involved in the regulation of biotic and abiotic stress responses and a more detailed analysis of the functional role of these two genes was conducted through overexpression studies in transgenic *Arabidopsis*. Our results provide new information on the role of MAPK cascade proteins in plant response to stress.

## Results

### Molecular characterization of VvMKK family genes

The availability of the grapevine genome sequence allowed us to identify the MAPKK gene family members in grapevine. A total of five MAPKK genes were identified in the grapevine genome and designated as VvMKK1-VvMKK5. Because there is no standard nomenclature protocol for the previously-identified MAPKKs in *Arabidopsis*, these genes were named based on the coordinate order of them on grapevine chromosomes from top to bottom. The nomenclature, accession number, chromosomal localization, number of amino acids, gene length, molecular weight (MW), and isoelectric point (PI) of the identified VvMKK genes are listed in Table 1. The VvMKK genes are located on four chromosomes. VvMKK2 and VvMKK3 are located on chromosome 11 and VvMKK1, VvMKK4, and VvMKK5 are located on chromosome 9, 14, and 17, respectively. The ORFs of the VvMKK genes encode polypeptides ranging from 314 (VvMKK5) to 518 (VvMKK4) amino acids, ranging from 1169 bp (VvMKK5) to 6879 bp (VvMKK2) in length, with predicted molecular masses ranging from 34.94 KDa (VvMKK5) to 57.48 KDa (VvMKK4), with isoelectric point values ranging from 5.56 pI (VvMKK4) to 9.50 pI (VvMKK1) (Table 1).

**Table 1:** The characteristics of MAPKK genes in grapevine

Name	Gene model name	Chromosomal localization	AA length	Gene length	MW (KD)	PI
VvMKK1	VIT_09s0018g01820	chr9:19257820–19,263,704	420	5885 bp	46.81	9.50
VvMKK2	VIT_11s0016g01770	chr11: 1417490–1,424,368	354	6879 bp	39.15	6.00
VvMKK3	VIT_11s0016g02970	chr11: 2377706–2,381,306	354	3601 bp	39.85	6.02
VvMKK4	VIT_14s0066g00670	chr14: 27139067–27,144,949	518	5883 bp	57.48	5.56
VvMKK5	VIT_17s0000g01970	chr17:1537383–1,538,551	314	1169 bp	34.94	6.55

Columns 1–7 contain the protein acronym (Name), Vitis proteome Gene model name, chromosome location (Chr), protein length (Amino acid length), gene length, estimates of molecular weight (MW), and isoelectric point of the protein (PI) for each MAPKK (Mitogen-activated protein kinase kinase) gene are given

Full-length cDNA clones of all of the VvMKK genes were obtained by RT-PCR from ‘Pinot noir’ (PN40024) plants to confirm the results obtained from the whole genome sequence and to further classify the properties of the encoded proteins (Additional files 1: Table S1). Results of the cloning indicated that VvMKK4 had 99.94% similarity and VvMKK1, VvMKK2, VvMKK3, and VvMKK5 had 100% similarity with the sequences present in the whole genome sequence (Additional files 2: Figure S1).

The conserved motifs present in the translated proteins of all of the cloned grape MAPKK genes to corresponding orthologs in *Arabidopsis* were analyzed. A schematic representation of the analysis is presented (Additional files 3: Figure S2). The predicted peptides of all five VvMKK genes possess the three canonical motif structures for MAPKKs, as well as including the consensus sequence S/T-XXXXX-S/T.

The phylogenetic analysis of the five VvMKK genes separated them into four subgroups corresponding to subgroups in *Arabidopsis* (Additional files 4: Figure S3). VvMKK2 and VvMKK3 were placed in group A, along with three AtMKKs, while VvMKK4 was placed in group B, with only one paralogous member, AtMKK3. VvMKK1 was placed in group C and VvMKK5 was placed in group D when compared with *Arabidopsis* MAPKK sequences (Additional files 4: Figure S3). The exon/intron arrangement in MAPKK genes could also be divided into four subgroups based on their phylogenetic relationship (Additional files 4: Figure S3). Despite some differences in the length of particular exons, exon structural patterns in MAPKKs appear to be well conserved. For example, VvMKK2 and VvMKK3 in group A and located on same chromosome, exhibit high similarity and the same number of introns (7 introns) (Additional files 4: Figure S3). The *Arabidopsis* MAPKK genes in group C and D do not contain introns, although it appears that some introns have been gained during the evolution of the VvMKK1 gene in group C (Additional files 4: Figure S3). Collectively, five MAPKK genes were identified in grapevine.

### Expression of VvMKK genes

**Fig. 1:**
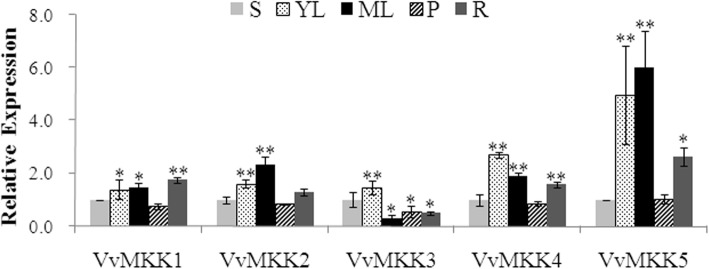
Relative gene expression of VvMKK genes in different grapevine tissues and organs as determined by qRT-PCR. The normalized relative expression in stem tissues was designated as having a value of “1”. S: stems; YL: young leaves; ML: mature leaves; P: petioles; R: roots. The data presented are the means ± SD of the relative expression of each gene as determined from three technical and three biological replicates. Bars represent SDs. ** and * indicated significant differences in comparison to stems at *P* < 0.01 and *P* < 0.05, respectively

The results of expression level of MAPKK genes in five different grapevine tissues and organs, indicated that VvMKK2 was highly expressed in young leaf blade and roots, relative to other tissue-types, while its closely-related sister gene, VvMKK3, was not expressed as highly as in these tissues (Fig. 1). VvMKK1, VvMKK4, and VvMKK5 were most highly expressed, in young and mature leaf blades, and roots in relation to in other tissue-types (Fig. 1). In general, the expression level of VvMKK genes was relatively higher in lamina tissues than in petioles.

**Fig. 2:**
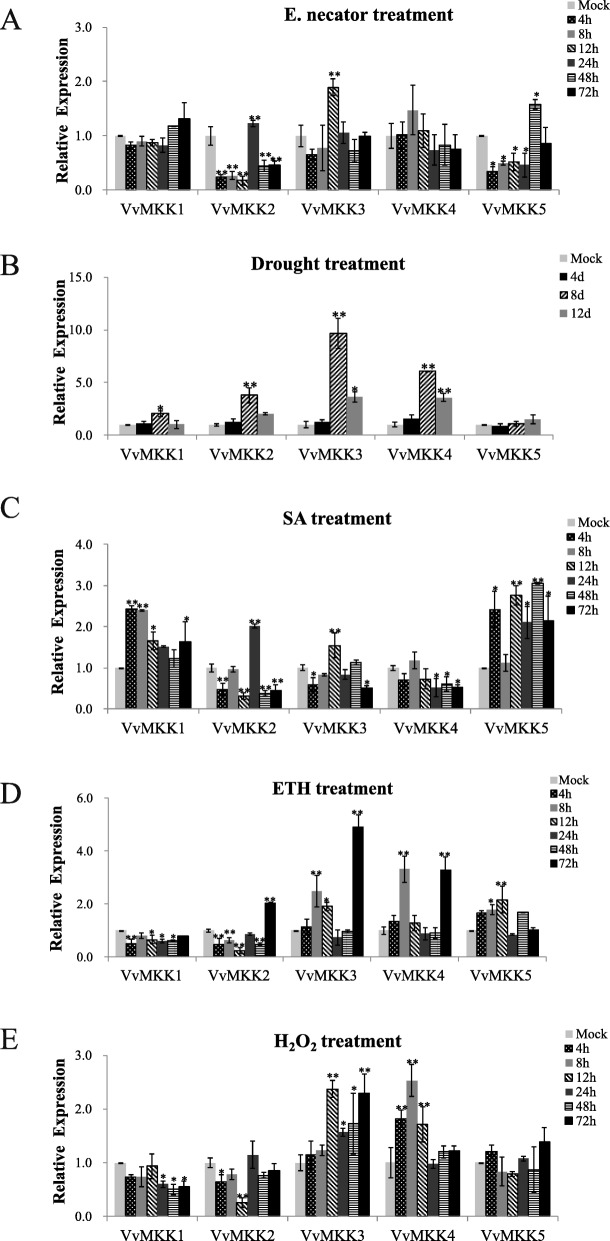
qRT-PCR analysis of VvMKK gene expression in grape leaves in response to biotic and abiotic stress treatments. **a**: *E. necator* (powdery mildew) infection; **b**: Drought treatment; **c**: Salicylic acid (SA) treatment; **d**: Ethanol (ETH) treatment; **e**: Hydrogen peroxide (H_2_O_2_) treatment (for details on treatments see [7]). Relative fold difference was determined in comparison to the normalized relative expression level of mock (untreated) tissues, which was designated as having a value of “1”

VvMKK3 exhibited the most rapid and greatest response to inoculation of leaves with powdery mildew where an evident up-regulation was detected at 12 h post-inoculation, after which expression gradually decreased (Fig. 2a). In contrast, VvMKK2 and VvMKK5 exhibited their highest expression at 24 h and 48 h post-inoculation, respectively (Fig. 2a).

Four VvMKK genes (VvMKK1 - VvMKK4) exhibited a similar and significant changes in expression in grapevine leaves in response to drought stress (Fig. 2b). Their expression level increased and peaked at 8 days after the onset of the drought treatment, after which their expression decreased. Notably, VvMKK3 and VvMKK4 exhibited a 5-fold increase in expression in response to the drought treatment (Fig. 2b). Regarding the response to SA, VvMKK1 and VvMKK5 exhibited the greatest increase in expression, relative to the untreated control (mock), while VvMKK4 was down-regulated, and VvMKK2 and VvMKK3 expression only increased slightly (one-fold) (Fig. 2c). The collective response of VvMKK genes in response to ETH and H_2_O_2_ was similar (Fig. 2d and e). In response to these treatments, VvMKK3 and VvMKK4 genes were dramatically up-regulated, relative to the untreated control, while VvMKK1 was down-regulated and VvMKK5 was significantly up-regulated by ETH but was unaffected by H_2_O_2_ (Fig. 2d and e).

### Over-expression of VvMKK2 in transgenic *Arabidopsis* enhances abiotic stress tolerance

Transgenic *Arabidopsis* lines overexpressing VvMKK2 were generated to evaluate its effect on abiotic stress tolerance. A total of 11 independent transgenic lines were selected based on kanamycin resistance and further confirmed by GUS detection (data not shown). Three lines (OE2, OE8, and OE9) homozygous for VvMKK2, which exhibited strong VvMKK2 expression in leaves as determined by GUS staining, were used in the stress tolerance assays, where their response was compared to wild-type (WT), non-transformed plants.

**Fig. 3:**
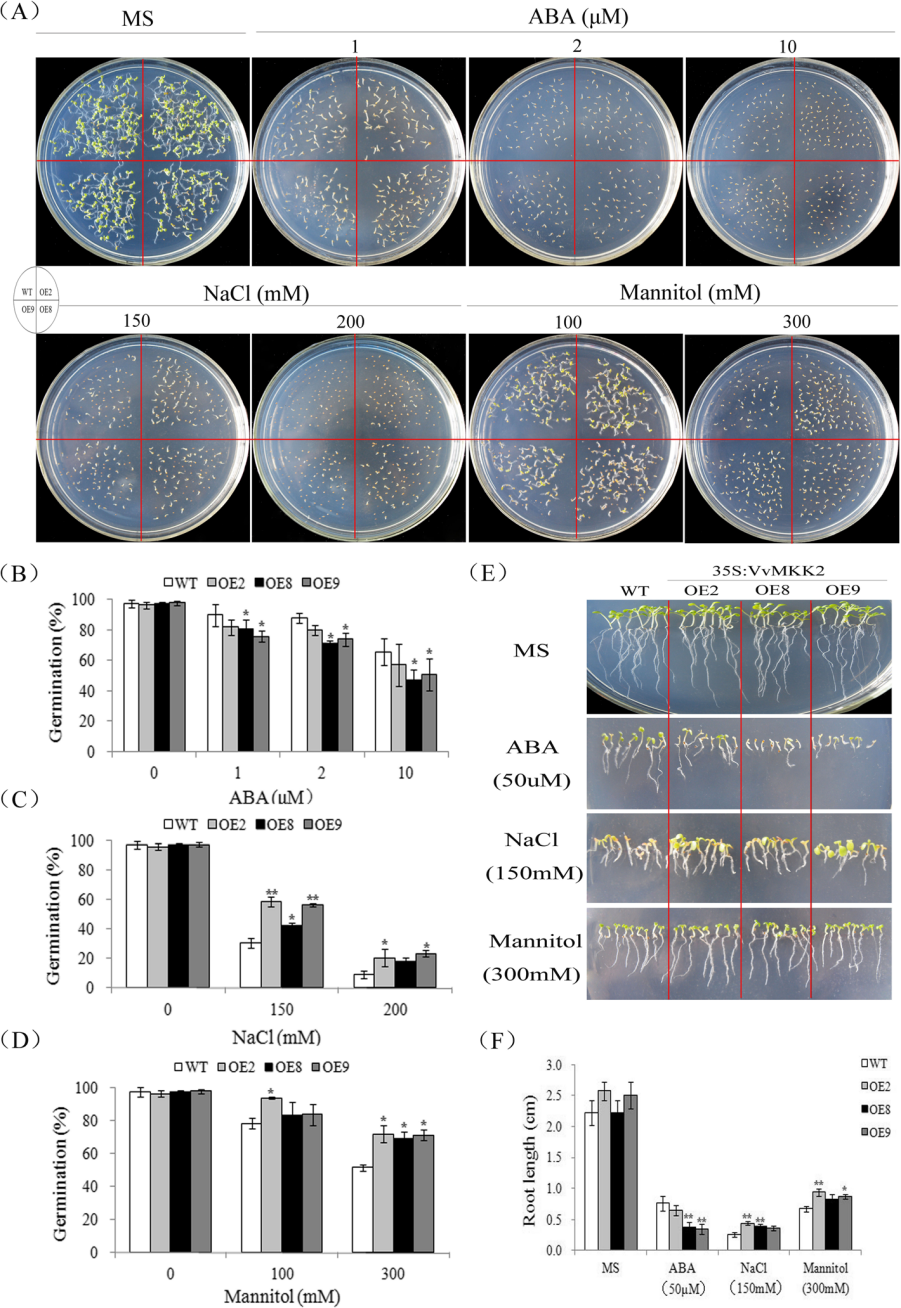
Osmotic stress tolerance of VvMKK2-transgenic *Arabidopsis* plants. **a**: Seed germination on 1/2 MS medium containing different concentrations of ABA, NaCl, and mannitol. **b**: Germination rate of WT and over-expressing (OE) lines in response to ABA. **c**: Germination rate of WT and OE lines in response to NaCl stress. **d**: Germination rate of WT and OE lines in response to mannitol stress. **e**: Seedling development in WT and OE lines on MS medium containing ABA, NaCl, or mannitol. **f**: Root length of in WT and OE seedlings after germination on MS medium

WT and VvMKK2-overexpressing transgenic (OE2, OE8, and OE9 lines) seeds germinated on 1/2 MS agar medium supplemented with different concentrations of ABA, NaCl, and mannitol (Fig. 3). As shown in Fig. 3a, no distinctive morphological differences were observed between WT and transgenic plants grown under normal, non-stress conditions (Fig. 3a). The germination rate of wild-type and transgenic seeds, however, were inhibited on normal 1/2 MS medium supplemented with various concentrations of ABA and no significant differences in the rate of germination was observed between WT and transgenic seeds with increasing concentrations of ABA (Fig. 3a and b).

When transgenic and wild-type *Arabidopsis* seeds were exposed to 150 mM NaCl, germination rates were approximately 40% in WT seeds and 60% in transgenic seeds overexpressing VvMKK2. When WT and transgenic seeds were exposed to 200 mM NaCl, germination rates dropped to 10 and 20%, respectively. These results indicate that over-expression of VvMKK2 enhance seed tolerance to salt-induced inhibition of germination (Fig. 3a and c). On a medium containing 100 mM mannitol, however, no significant difference was observed between the germination of WT and transgenic seeds overexpressing VvMKK2. Notably, at 300 mM mannitol differences in germination rates were significant where the germinations rates were about 70% and ~ 50% for transgenic and WT seeds, respectively (Fig. 3a and d).

The effect of ABA, NaCl, and mannitol on root growth in transgenic and WT plants was further examined. No significant differences in root length were observed when no amendments were added to the growth medium. Root growth was inhibited to a greater degree in transgenic plants than in WT plants when the growth medium was supplemented with 50 μM ABA (Fig. 3e and f). Transgenic plants exposed to 150 mM NaCl, however, exhibited significantly longer roots than WT plants under the same conditions, and transgenic plants also exhibited larger cotyledons (Fig. 3e and f). When exposed to 300 mM mannitol, root growth of WT seedlings was severely inhibited, while root growth in transgenic plants was only slightly affected (Fig. 3e and f).

**Fig. 4:**
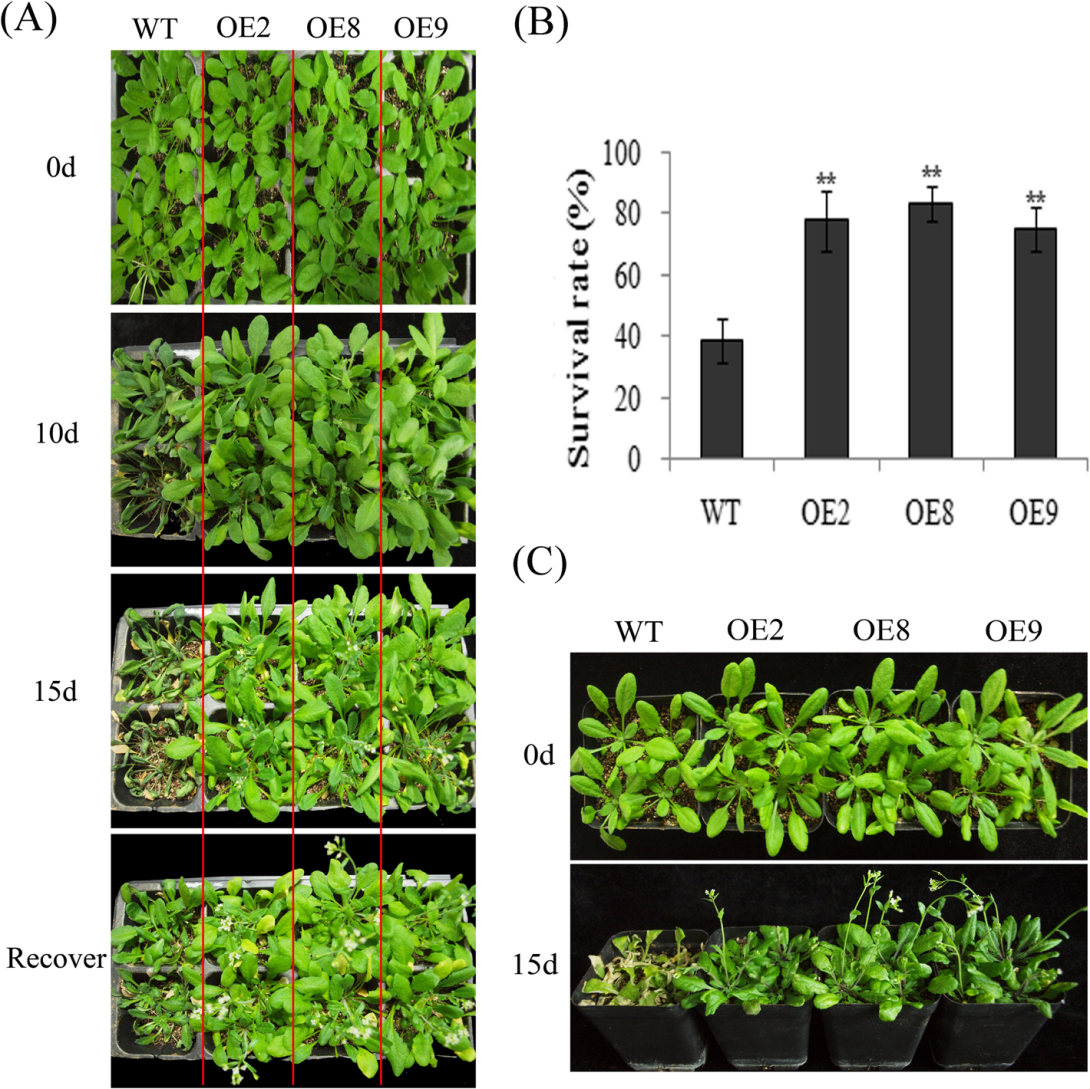
Over-expression of VvMKK2 enhances drought and salt tolerance in transgenic *Arabidopsis*. **a**: Phenotype of plants treated with drought stress. **b**: The survival rate in overexpressing (OE) lines after 2 days of re-watering following the drought treatment (withholding water for 20 d). **c**: Phenotype of plants subjected to salt stress

WT and transgenic plants were deprived of water for 20 d, followed by re-watering for 2 d, to assess the effect of VvMKK2 over-expression on drought tolerance. Results indicated that leaf wilting was more evident in WT plants than in transgenic plants after 20 d without water. Transgenic plants overexpressing VvMKK2 also recovered their growth more rapidly than WT plants when plants were re-watered (Fig. 4a). Transgenic lines exhibited an 80% survival rate when evaluated after 3 days of re-watering, while WT plants exhibited only a 40% survival (Fig. 4b). These results indicate that over-expression of VvMKK2 enhances drought tolerance in *Arabidopsis.*

WT and transgenic plants growing in the same pot were irrigated with 150 mM NaCl solution for 15 d to determine if the increases salt tolerance during germination and root growth by over-expression of VvMKK2 was also present during vegetative growth. Although both WT and transgenic plants showed severe inhibition of growth occurred when exposed to the salt stress compared to growth under non-stress conditions, transgenic plants showed less severe inhibition (Fig. 4c). Additionally, most of the transgenic plants even survived under high salinity conditions, with some exhibiting flowering, while leaves in WT plants became wilted, curled, and even died over time (Fig. 4c). These data indicate that over-expression of VvMKK2 improves salt stress tolerance in transgenic *Arabidopsis* plants during both seed germination and vegetative growth.

### Over-expression of VvMKK4 in transgenic *Arabidopsis* enhances salt stress tolerance

VvMKK4 was transformed into *Arabidopsis* plants to determine the effect of VvMKK4 over-expression on plant tolerance to multiple stresses. A total of 9 independent transgenic lines were obtained by kanamycin resistance selection and subsequently confirmed by GUS detection. Three VvMKK4 homozygous lines (OE3, OE7, and OE8) in which GUS was highly expressed in leaves were used for the stress tolerance tests.

**Fig. 5:**
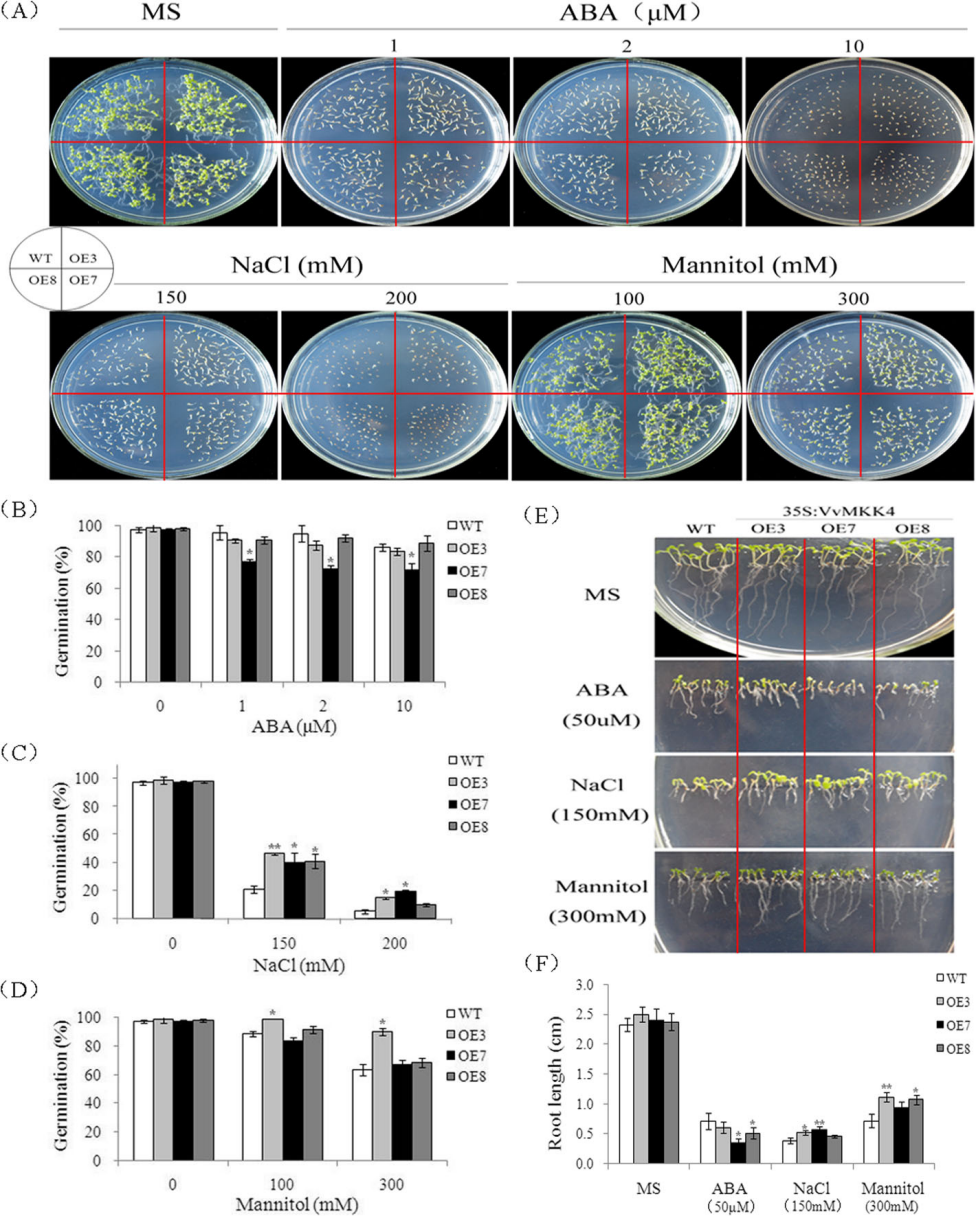
Osmotic stress tolerance of VvMKK4-transgenic *Arabidopsis* plants. **a** Seed germination in wild-type (WT) and overexpressing (OE) seeds on 1/2 MS medium supplemented with different concentrations of ABA, NaCl, or mannitol. **b**, **c** and **d** Germination rate of WT and OE seeds in A. **e** Seedling development of WT and OE lines grown on MS medium supplemented with ABA, NaCl, or mannitol, **f** Root length in WT and OE seedlings after germination on MS medium

The germination rate of seeds from WT and three independent transgenic lines (OE3, OE7, and OE8) were examined in response to a variety of abiotic stresses (Fig. 5). Results indicated that WT and transgenic lines did not exhibit any significant differences in the rate of germination when seeds were placed on MS medium supplemented with 50 μM ABA (Fig. 5a and b). On MS medium supplemented with 150 mM NaCl, a 40% germination rate was obtained for transgenic seeds compared to a 20% germination rate for WT seeds (Fig. 5a and c). Germination rates on 200 mM NaCl were 20% for transgenic seeds and only 5% for WT seeds (Fig. 5a and c). The difference in the germination rate of WT and transgenic seeds plated on MS medium supplemented with 300 mM mannitol was not significant, except for the OE3 transgenic line (Fig. 5a and d).

The effect of ABA, NaCl, and mannitol treatments on root length was also assessed in WT and transgenic plants. Root length was not significantly different between transgenic and WT plants growing on normal MS medium without any of the amendments (Fig. 5e and f). In contrast, root length was shorter in the transgenic plants than in WT plants when the MS medium was supplemented with 50 μM ABA (Fig. 5e and f). As with transgenic VvMKK2 plants, root length was longer in VvMKK4 transgenic *Arabidopsis* plants than in WT plants growing on MS medium supplemented with 150 mM NaCl (Fig. 5e and f). The transgenic plants growing under salt stress also had larger cotyledons. Roots were also longer in plants of all the transgenic lines overexpressing VvMKK4 than in WT seedlings growing on MS medium supplemented with 300 mM mannitol (Fig. 5e and f).

**Fig. 6:**
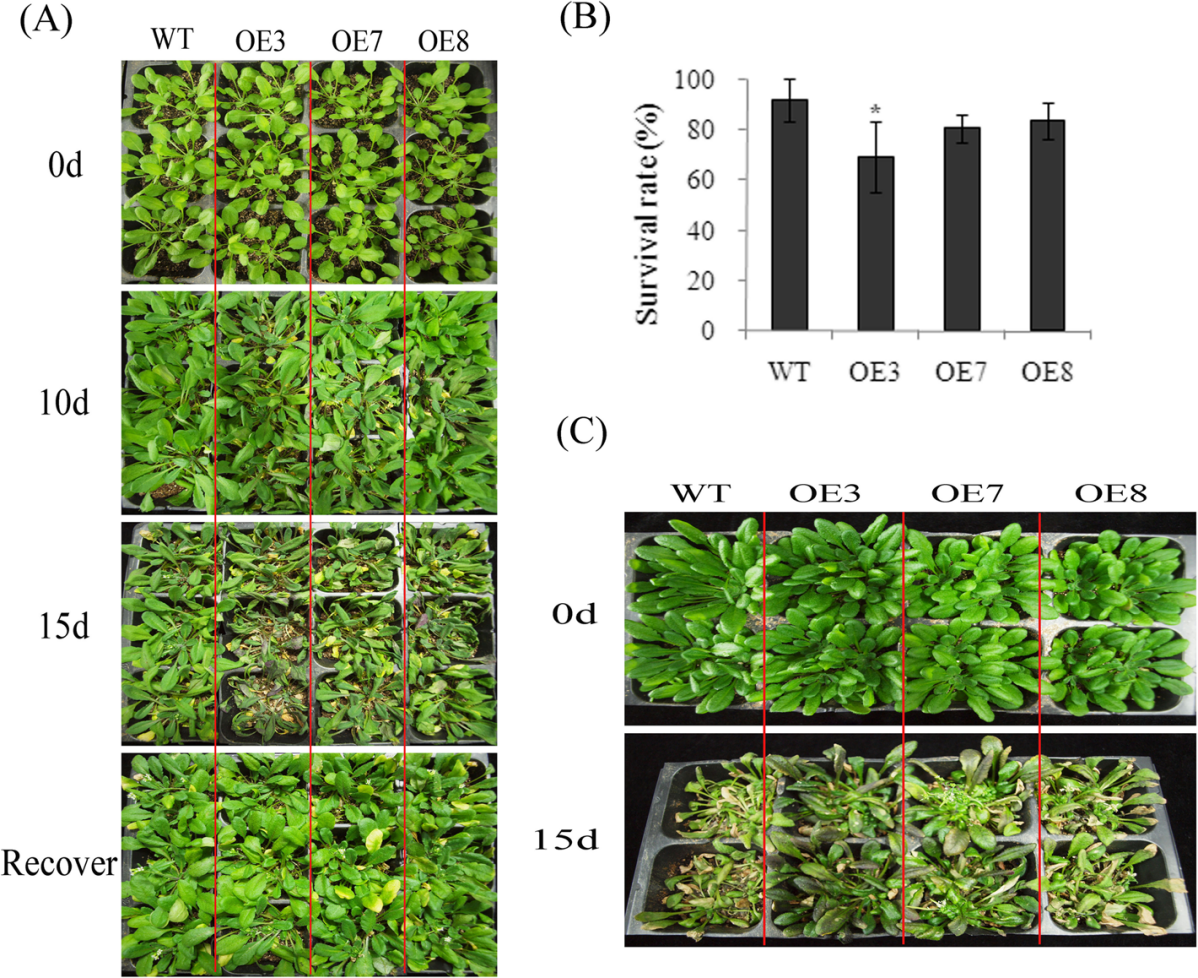
Over-expression of VvMKK4 enhances drought and salt tolerance in transgenic *Arabidopsis*. **a**: Phenotype of plants subjected to drought stress (withholding water for 20 d). **b**: Survival rate in wild-type (WT) and overexpressing (OE) lines after 2 d of re-watering following a drought stress treatment (withholding water for 20 d). **c**: Phenotype of plants subjected to salt stress

The performance of VvMKK4 over-expression in *Arabidopsis* plants under drought and salt conditions was also evaluated. WT and transgenic plants were deprived of water for 20 d, followed by re-watering for 2 d, to simulate drought stress and recovery. Leaves of both WT and transgenic lines exhibited wilting after 20 d of withholding water, however, the level of wilting was not as extensive in the transgenic plants as it was in the WT plants (Fig. 6a). After watering was resumed, most of transgenic and WT plants recovered their growth, and no significant differences in the survival rate between the two plant types (transgenic and WT) were observed, with both exhibiting an 80% survival rate (Fig. 6a and b). The tolerance of transgenic and WT plants to high levels of salinity was also examined (Fig. 6c). Growth was significantly reduced in both WT and transgenic plants; however, WT plants were impacted to a greater extent, exhibiting a higher level of wilting and chlorosis in response to the salt stress (Fig. 6c).

## Discussion

### Identification, characterization, and isolation of VvMKK genes

The MAPKKK-MAPKK-MAPK signaling cascade plays an important role in regulating developmental processes and in the transduction of environmental signals [4, 14]. MAPK signaling-related genes have been characterized in many plant species through an analysis of their whole genome sequence. This includes MAPKK family genes, which have been systematically investigated in *Arabidopsis* [11], rice [35], tomato [18], maize [8], apple [36], the monocot grass species, *Brachypodium distachyon* [37] and others. A previous study in *Arabidopsis* demonstrated that MAPKK proteins from the same subgroup or clade tend to cluster together, and that most MAPKK genes generally cluster in well-resolved clades, except for the AtMKK10 clade. The phylogenetic classification of VvMKK genes/proteins is also supported by an analysis of their conserved motifs (Additional files 3: Figure S2) and gene structure (Additional files 4: Figure S3). Similar to other aligned MAPKs, VvMKKs possesses a consensus sequence of the signature motif VGTxxYMSPER, the plant-specific phosphorylation target site motif, −S/TxxxxxS/T-, and the conserved aspartic acid and lysine residues within the active site motif (−D (L/I/V) K-). Conserved motif analyses revealed that all the VvMKK proteins contain the conserved characteristics and each subfamily shared similar motifs reported in other plant species, such as *Arabidopsis*. Interestingly, MAPKKs of subgroups A, C and D encode relatively short proteins, while AtMKK3 and VvMKK4 of subgroup B have an unusual structural feature consisting of a nuclear transport factor (NTF) domain in the extended C-terminus region. This chimerical arrangement has been reported to have had a long evolutionary history in the lineage of photosynthetic eukaryotes [38]. Gene structure analysis also indicated that MAPKK gene members within the same subgroup possess a similar exon-intron organization. Despite some modest differences in the length of particular exons, the exon structural pattern of MAPKKs, including VvMKKs, appears to be well conserved. For example, VvMKK2 and VvMKK3 of group A, located on same chromosome, had the highest similarity to each other and both had seven introns (Additional files 4: Figure S3). *Arabidopsis* MAPKK genes in group C and D do not have introns, while some gain in introns appears to have occurred during the evolution of the VvMKK1 gene in group C (Additional files 4: Figure S3). Overall, the conserved motif and gene structure analyses indicate that MAPKK genes in the same group exhibit similar conserved motifs and exon-intron organization, suggesting that MAPKKs within the same subgroup have been closely-related to each other.

The number and size of MAPKKs in grapevine was found to be relatively small and it appears that grapevine has the lowest number of MAPKK genes among plant species in which MAPKK genes have been identified (Additional files 5: Table S2). While the genome of grapevine may actually contain fewer MAPKK genes than other plant species, it is also possible that additional MAPKKs may exist in portions of the published grape genome containing gaps [39].

### VvMKK expression in response to various stress conditions

A high level of expression of a gene in a specific tissue, relative to other tissues, usually indicates that the gene plays a functional role [40]. In the current study, most of the VvMKK genes were expressed higher in leaf tissue than in any of the other examined tissues/organs (Fig. 1). Higher expression of a specific VvMKK gene may be related to its role in the development of the corresponding organ or indicate the site where the protein perceives and translates cellular signals. The different expression pattern of VvMKK2 compared to its closely-related, duplicated sister gene, VvMKK3, indicates that even though duplicated genes have a high degree of similarity in amino acid sequences, one cannot extrapolate that they should also have a similar function or are involved in the same signaling pathway. In fact, the organ-specificity of VvMKK2 and VvMKK3 expression varied, presumably based on their function rather than their sequence similarity [37]. Additional research will be required to definitively determine the function of the various MAPKK family genes in grapevine.

Conducting a survey of a gene family has been an effective strategy for identifying candidate genes or specific promoters involved in a particular biological process [41]. Accumulating evidence indicates that MAPKK proteins are involved in plant response to a variety of biotic and abiotic stresses [38]. In the current study in grapevine, several VvMKKs were found to be activated by several of the tested stress-related treatments and exhibit different patterns of expression (Fig. 2). The potential involvement of specific VvMKK genes in the response to different stress stimuli is highly likely as MAPKK are known to participate in a finely-tuned network of signal transduction cascades in plant cells. Thus, different MAPKK genes would be involved in the regulation of different developmental processes or stresses. Genes from different plant species having similar regulatory functions was also indicated in the cluster analysis but it remains to be determined if there is a correlation between gene classification and function [42]. MAPKK genes in groups A and B were reported to be mainly involved in abiotic stress response in *Arabidopsis*. For example, AtMKK1 has been associated with drought signaling [43], and AtMKK2 has been reported to play a role in abiotic stress tolerance [25]. The phylogenetic tree constructed in the present study determined that VvMKK2 belongs to group A, which also contains AtMKK1, AtMKK2 and AtMKK6. Further analysis indicated that VvMKK2 was up-regulated in response to powdery mildew, drought, SA, and ETH treatments, and particularly by the drought treatment. This finding is almost identical to previous study [22]. AtMKK3 belongs to the B subfamily along with VvMKK4 which was demonstrated to be associated with abiotic stress (such as salt stress) response, as well as plant growth and development. The present study also demonstrated that VvMKK4 can be induced by drought, ETH, and H_2_O_2_. Based on the conducted experiments, VvMKK2 and VvMKK4 genes exhibited a consistent and significant increase in expression in response to several stress-related treatments and most specifically to the drought treatment. Thus, they were selected for further analysis by over-expression studies in *Arabidopsis*.

### Over-expression of VvMKK2 and VvMKK4 in transgenic *Arabidopsis* enhances stress tolerance

VvMKK2 and VvMKK4 were transformed and overexpressed in *Arabidopsis* to further determine their role in stress response. *Arabidopsis* was selected as the target for transformation due to the inherent difficulty of grapevine transformation. Results indicated that over-expression of VvMKK2 and VvMKK4 improved stress tolerance in transgenic *Arabidopsis* plants as evidenced by their germination rate, root length, and survival rate after being subjected to a variety of stress treatments (Figs. 3, 4, 5 and 6). ABA is an important regulator of plant response during seed germination and early seedling development [44]. Results indicated that no significant differences were present between the measured parameters in WT and over-expressing *Arabidopsis* lines when they were grown on normal MS medium without any supplements, however, seed germination in both were inhibited when the MS medium was supplemented with 50 μM ABA but the level of inhibition was greater in the transgenic rather than the WT seeds. Further experiments indicated that the over-expressed genes conferred tolerance to different abiotic stresses. VvMKK2-overexpression plants exhibited increased salt and drought stress tolerance, while VvMKK4-overexpression plants exhibited increased tolerance to salt stress. These results are consistent with previous studies on the effect of MAPKK genes on salt stress tolerance [45]. The differential tolerance of these transgenic plants to different stresses, may reflect the specificity of each of the VvMKK proteins on the regulation of downstream genes. Further physiological and genetic experiments will be required to validate and further elucidate the function of these genes.

### Possible modules of MAPKKs and MAPKs involved in grapevine interactions

Plant MAPKs have been well documented to regulate various physiological responses by interacting with upstream and downstream protein components in a cascade manner MAPKs, MAPKKs and MAPKKKs [46]. To date, we have identified12 VvMAPKs, 5 VvMKKs and 45 VvMAPKKKs in grapevine [7, 33], further studies are needed to elucidate the mechanisms involved in stress tolerance in grapevine and the pair-wise interactions of MAPKKK-MAPKK-MAPK genes. It is well known that the modules of AtMKK1/AtMKK2-AtMAPK4 negatively regulates immunity in *Arabidopsis* [23]. VvMKK2 is a putative ortholog of *Arabidopsis* AtMKK1 and AtMKK2, and it was up-regulated in response to powdery mildew, drought, SA, ETH treatments in this study, particularly by the drought treatment, therefore, its function was ectopically verified in resistance to salt and drought stresses in *Arabidopsis*. VvMAPK9 which is closely related to AtMPK4 also was up-regulated in response to powdery mildew, drought, SA, ETH treatments [7]. Thus, it is possible that VvMKK2 and VvMPK9 form a functional MAPK cascade, suggesting that the VvMKK2 - VvMPK9 interactions might be involved in salt and drought stress as well as defense against pathogens. Further comprehensive analysis of protein-protein interactions among VvMKKs and VvMAPKs will be helpful to establish the MAPK cascades and their signaling networks.

## Conclusions

In this study, we identified 5 MAPKK genes from grapevine, which were unevenly distributed in 4 chromosomes and were classified into 4 clades, which were identical to the *Arabidopsis* MAPKK genes. The gene expression analysis revealed that most of the genes were expressed in different tissues and in response to powdery mildew, drought, SA, ETH stresses. Overexpression of VvMKK2 in *Arabidopsis* improved salt and drought stress tolerance while overexpression of VvMKK4 only improved salt stress tolerance. Further studies are needed to elucidate the mechanisms involved in stress tolerance in grapevine and the pair-wise interactions of MAPKKK-MAPKK-MAPK genes. The results from the study would be useful for breeding grape cultivars with improved abiotic stress tolerance, also provide knowledge that could be used to manipulate the expression of these genes for improved stress tolerance.

## Methods

### Plant material, growth conditions, and stress treatments

PN40024 (*V. vinifera*, an inbred line of ‘Pinot Noir’) grapevine were maintained in vitro on 1/2 MS medium supplied with 0.3 mg/L 3-indolebutyric acid (IBA, Sigma, USA), under a 16/8 h photoperiod (100 μmol m^− 2^ s^− 1^) at 25 °C in the growing chamber. The expression pattern of VvMKK genes in response to biotic (powdery mildew) and abiotic (drought) stress conditions, as well as hormone and chemical treatments (SA, ETH and H_2_O_2_) also was investigated by qRT-qPCR as previously described [7, 33]. Tissue-specific expression was analyzed in five tissue-types obtained from in vitro (tissue culture) plants. Stems, mature leaves (sixth and seventh nodes); young leaves (first and second nodes), leaf petioles, and roots were harvested separately, frozen in liquid nitrogen, and stored at − 80 °C until they were used in subsequent analyses.

Seeds of *Arabidopsis* ecotype Columbia (Col-0) and transgenic plants were surface sterilized by soaking them in 75% ethanol (v/v, 1 ml) for 5 min and in 5% NaClO (v/v, 1 ml) for 20 min. Seeds were then rinsed 3–5 times with sterile distilled water and plated on solidified 1/2 MS medium containing 3% (w/v) sucrose. The plated seeds were first incubated for 2 days at 4 °C in the dark before being placed and maintained at 22 ± 1 °C under a 16/8 h of light/dark cycle and 80% RH. Then we transplanted *Arabidopsis* with full-length cotyledons onto pots containing sterilized substrate (vermiculite: nutrient soil: perlite = 9: 3: 1), and each pot contained 4 seedlings, cover with plastic wrap. When the seedlings grow to 2–4 leaves, remove the plastic wrap and cultivate normally until the stress treatment.

PN40024 plants were kindly provided by Dr. Anne-Françoise Adam-Blondon, INRA, France. The *Arabidopsis* seeds were obtained from the Laboratory of Fruit Tree Biotechnology of Nanjing Agricultural University (Nanjing, Jiangsu Province, China). We declare that the collection of plant materials complies with institutional, national, or international guidelines.

### Identification, cloning, and analysis of grape MAPKK genes

To identify members of the MAPKK gene family in grapevine, *Arabidopsis* MAPKK protein sequences [47] retrieved from the TAIR10 (http://www.arabidopsis.org/) database were used as a query to search against the *Vitis vinifera* Proteome database (12X V1) (http://genomes.cribi.unipd.it/grape/) [34] and NCBI databases (http://www.ncbi.nlm.nih.gov/) using a profile Hidden Markov Model-based search (HMMER: http://hmmer.wustl.edu/). A MEME (Multiple Expectation maximization for Motif Elicitation) (http://meme.nbcr.net/meme/cgi-bin/meme.cgi) analysis was conducted on the predicted candidate proteins of the MAPKK gene family in grapevine. Each retrieved sequence was subsequently examined for conserved MAPKK signature motif sequences. MAPKK gene models were only accepted if they displayed the consensus sequences for dual-specificity protein kinases, including the conserved motif (−D (L/I/V) K-), the plant-specific phosphorylation target site motif (−S/TxxxxxS/T-), and the signature motif (VGTxxYMSPER) [11].

Full-length clones of the grape MAPKK genes were obtained by RT-PCR combined with the rapid amplification of cDNA ends (RACE) method. Gene-specific primers were designed based on the predicted MAPKK gene sequences. The primer pairs designed to amplify ORFs and 3′ untranslated region sequences are listed (Additional files 6: Table S3 and Additional files 7: Table S4, respectively). The PCR protocol used was as previously described [7]. After confirming the accuracy of the obtained full-length sequences, multiple sequence alignments of predicted VvMKKs sequences with cloned VvMKK coding sequences was conducted using the ClustalW program at the nucleotide level. The phylogenetic trees and exon/intron structures of MAPKKs in *Arabidopsis* and grapevine were constructed as previously described [7, 33].

### RNA extraction and qRT-PCR expression analysis

Total RNA was extracted from the collected samples with a method modified from the previously described [48]. First strand cDNA synthesis was carried out using 1 μg total RNA and a PrimeScritpt RT reagent Kit (TaKaRa, Japan) following the manufacturer’s instructions. Reverse transcription - quantitative PCR (qRT-PCR) was performed as previously described [7, 33]. The gene-specific primers used in the qRT-PCR analysis of MKK genes were designed using Beacon Designer 7.0 software (Premier Biosoft International, USA) and targeted the 3′ UTR of each of the genes and designed to generate approximately 200 bp products (Additional files 8: Table S5). Grapevine actin gene was selected as an internal control to normalize the total amounts of cDNA present in each reaction. The relative expression levels of the target genes were assessed using the 2^-ΔΔCt^ method, where ΔΔCt = (Ct_target gene_ - Ct_actin_) _treatment_ - (Ct_target gene_ - Ct_actin_) _control_ [7]. Each sample comprised three biological replicates and the analysis represented the results of three independent experiments.

### Transformation of *Arabidopsis*

Full-length cDNA of MAPKK genes (VvMKK2 and VvMKK4) was amplified by PCR using primers that included *BamH*I and *Sac*I restriction sites on their respective 5′-ends (Additional files 9: Table S6). The PCR-generated fragment was fused to the GUS reporter gene in a binary vector pCAMBIA1301. The inserted gene was placed under the control of the *Cauliflower mosaic virus* 35S (CaMV 35S) promoter. After sequence confirmation, the construct was introduced into *Agrobacterium tumefaciens* strain EHA105. *Arabidopsis* plants (Col-0 ecotype) were transformed using the floral-dip method [49]. Transgenic seedlings were selected on 1/2 MS medium containing 50 mg/L kanamycin and further confirmed by PCR. Two week-old seedlings were used for GUS histochemical staining assays as previously described [50]. T3 progeny of transgenic *Arabidopsis* were used for the stress-tolerance assay.

### Seed germination assay and measurements of root growth

Surface-sterilized seeds from the T3 transgenic lines and wild-type (WT) plants were placed on 1/2 MS plates supplemented with various concentrations of mannitol (100 mM, 300 mM) or ABA (1 μM, 2 μM, 10 μM), or NaCl (100 mM, 200 mM), among them the mannitol is used to mimic water stress in vitro cultures [51]. The seeds were stratified by incubation in the dark at 4 °C for 3 days prior to placing them in the light. The number of seeds that germinated was expressed as a percentage of the total number of seeds plated. Germination rates were scored for 15 days and plants were photographed. Three replicate plates were used for each treatment.

For the root growth assay, transgenic and WT seeds were placed on 1/2 MS agar plates for germination. Two days after the seeds germinated, seedlings from each line were transferred carefully to a new 1/2 MS agar plate with or without 50 μM ABA, 300 mM mannitol, or 150 mM NaCl. The plates were placed vertically on a rack and root length in each of the seedlings was measured after 7 days of growth. The experiment was repeated at least three times.

### Stress-tolerance assessment of WT and transgenic plants

For the drought treatment, seeds of transgenic and wild-type plants were germinated and grown on 1/2 MS medium for 2 weeks, and then transplanted into plastic pots and allowed to grow for 5 weeks while being watered regularly. The drought treatment was commenced by withholding water. To assess survival, plants grown in the plastic pots were not watered for 20 d, followed by 3 days of re-watering after which survival was assessed and the plants were photographed. Survival rate was calculated as the number of surviving plants/total number of experimented plants × 100. Plants exhibiting > 50% green tissue were considered as having survived.

For the salt tolerance assays, *Arabidopsis* seedlings were cultured as described above. Water was withheld and then plants were irrigated with 300 mM NaCl solution, which was administered from the bottom of the pot for 15 d. Changes in the plant appearance were observed over that period of time. The drought and salt assays were performed in triplicates.

### Statistical analysis

Statistical significance of the various treatments was assessed by analysis of variance (ANOVA) and significant differences in treatment means between transgenic and WT plants were determined using a Fisher’s LSD test, at a significance level of *P* < 0.05 and *P* < 0.01. All statistical analyses were conducted using SAS software (version 8.0, SAS Institution, NC, USA). Photographs were prepared using Photoshop CS5 (Microsoft and Adobe, USA).

## Supplementary information

**Additional file 1 **: **Table S1.** The nucleic acid sequence of cloned VvMKK genes.

**Additional files 2 **: **Figure S1.** Alignment of nucleic sequences of each of the cloned VvMKK genes and the corresponding sequence of the predicted gene in the grapevine whole genome sequence database. Sequence homologies are highlighted in black while differences are highlighted in grey.

**Additional files 3 **: **Figure S2.** Alignment of amino acid sequences and domain analysis of MAPKK genes from grapevine and *Arabidopsis*. Alignment was performed using ClustalW program. The red-box indicates the conserved signature motif, the green-box indicates the plant MAPKK specific motif -T/SXXXXXS/T- and the active site -D (I/L/V) K- motif is highlighted in blue.

**Additional files 4 **: **Figure S3.** Phylogenetic relationship and schematic diagram of intron/exon structure of MAPKK genes in grapevine and *Arabidopsis*. The phylogenetic tree (left panel) was created using MEGA5.0 software with the neighbor-joining (NJ) method. Bootstrap values for 1000 replicates are indicated at each branch. Letters A-D on the right indicate different groups of MAPKKs. Exon/intron structures of the MAPKK genes are shown in the right panel. The green boxes indicate exons, while the single lines indicate introns. UTRs are indicated by thick blue lines at both ends. 0, 1 and 2 represented different intron phases. Gene models are drawn to scale as indicated on the bottom of the figure.

**Additional files 5 **: **Table S2.** The number of MAPKK gene family members in different plant species.

**Additional files 6 **: **Table S3.** The primer sequences used to clone the MAPKK genes in grapevine by PCR.

**Additional files 7 **: **Table S4.** The primer sequences used for 3′ RACE of the MAPKK genes in grapevine.

**Additional files 8 **: **Table S5.** The primer sequences of the MAPKK genes in grapevine for quantitative RT-PCR.

**Additional files 9 **: **Table S6.** The primer sequences used to insert VvMKK2 and VvMKK4 genes into transformation vectors.

## Data Availability

The datasets supporting the conclusions described in this article are included within the manuscript and its additional files. Other data are available from the corresponding authors on reasonable request. The sequences of cloned five VvMKK genes were submitted to the NCBI database with the with the link of https://www.ncbi.nlm.nih.gov/nuccore, under the accession number MT154080, MT154081, MT154082, MT154083 and MT154084, respectively.

## Abbreviations

AA: Amino acids
ABA: Abscisic acid
BLAST: Basic Local Alignment Search Tool
bp: Base pair
CaMV35S: 35S promoter of cauliflower Mosoic virus
cDNA: Complementary Deoxyribonucleic Acid
*E. coli*: *Escherichia coli*
ERK kinase: Mitogen-activated protein kinase
ETH: 2-chloroethyl phosphonic acid (Ethephon)
H_2_O_2_: Hydrogen peroxide
MAPK: Mitogen-activated protein kinase
MAPKK: Mitogen-activated protein kinase kinase
MAPKKK: Mitogen-activated protein kinase kinase kinase
MAPKKKK: Mitogen-activated protein kinase kinase kinase kinase
MAP 3 K: Mitogen-activated protein kinase kinase kinase/MAPKKK
MAP 4 K: Mitogen-activated protein kinase kinase kinase kinase/MAPKKKK
MEK: Mitogen-activated protein kinase kinase/MAPKK
MEKK: Mitogen-activated protein kinase kinase kinase/MAPKKK
MKK: Mitogen-activated protein kinase kinase/MAPKK
MPK: Mitogen-activated protein kinase/MAPK
MS: Murashige and Skoog medium
MW: Molecular weight
OE: Over-expression
PM: Powdery mildew
PI: Isoelectric point
RACE: Rapid amplify cDNA end
SA: Salicylic acid
SIMK: Salt-stress-inducible MAPK
SIMKK: Salt-stress-inducible MAPKK
SIPK: SA-induced protein kinase
TEY: Threonine-glutamic acid-tyrosine
3′ UTR: 3′ untranslated region
WIPK: Wound-induced protein kinase
WT: Wild type
