# Supplementary material for: Cloning, molecular and functional characterization by overexpression in Arabidopsis of MAPKK genes from grapevine (Vitis vinifera)
Source: BMC Plant Biol. 2020 May 7;20:194. doi: 10.1186/s12870-020-02378-4 (PMC7203792; doi:10.1186/s12870-020-02378-4)
Supplement: Supplementary file 1 — Additional file 1 : Table S1. The nucleic acid sequence of cloned VvMKK genes. [file 12870_2020_2378_MOESM1_ESM.docx]

Table S1. The nucleic acid sequences of the cloned genes. The black colour represent 5’ and 3’ untranslated regions, red colour denote the open reading frames (ORFs) of VvMKK genes.

>VvMKK1 (5′-3′)

AGCTCTTTTCACAAATCAGACAGTTCTCTCTTCTTCCAAGAACCTTCTCTCTTCTTTTCTCTCTCTAGAAAGACTCTTTCCATGGATGATTTCCTCAAGACGATCATCCAACGCCAGAACCCCCTCTTCTTCCTCGTACCCATGAAACCACAGCCACAAATCCCACCCTTTCTCTCTCCAGTTTCTTGCTAACGAAGCATACCCCTCCCTGCAAAACAGAAATATAACAAGCCCCAGAAAGGAAAACAAGAAAAGGGAAAGAGACCCAACTCAGTCGTCCACTCATGAAGCCGAATCAACCCCCTACCGCCGCCGCCTCCTCCTCAGCCGCCAATACCCGGATTCGGCCTCGACGGCGGCCCGACCTCACGCTTCCTCTCCCGCAGCGGGATCCTTCCCTTGCCGTGCCGCTTCCTCTTCCGCCGACGTCGGCTCAGTCTTCGTCCTCCGGAGGGGCGGCGGCGGCGGCGACGCCAGCGTTCTCGGAGCTGGAGAGAATCAACAGAATCGGAAGCGGTAGCGGCGGCACCGTGTACAAGGTCCTCCACAGGCCGACGGGGAGGTTGTACGCCTTGAAGGTGATCTACGGAAACCACGACGACGACGTGCTCCGCCAGATCTGTCGAGAAATCGAGATCCTCAGAGATGTGGATAACCCCAACGTGGTGAAGTGCCACGACATGTACGACCACGCCGGCGAAATCCAGGTGTTGCTGGAGCACATGGACGGTGGATCTCTTGAGGGGACTCACATCGCGGACGAGCTCGCTCTCTCCGATCTCGCGTTTCAGATCCTCTCCGGCCTCCACTACCTCCATCGCCGGAAAATCGTGCACCGCGACATCAAACCTTCGAACCTCCTAATCAACGCCAGGCGACAAGTGAAGATCGCCGACTTCGGTGTGAGCCGGATCTTGGCTCAGACCATGGATCCGTGTAACTCATCGGTGGGAACGATCGCGTACATGAGTCCGGAGCGGATCAACACCGATCTCAACCACGGCAGATACGACGGCTACGCTGGCGACATCTGGAGCCTCGGTGTGAGCATCTTGGAGTTTTATTTAGGGCGTTTCCCGTTTGCTGTCGGCCGGCAAGGGGATTGGGCGAGTTTGATGTGCGCAATCTGTATGTCGCAGCCGCCGGAGGCGCCAGTCACTGCATCAAGGGAGTTTAGGGACTTCATATCACGGTGTCTGCAGAGGGATCCGGCGGTCCGGTGGTCGGCAGACAAACTTCTCCGGCACCCCTTTGTGCTTCAGAGTCAGAGAAGAAGAGGACAGAGTCAAAGCCAGGTTCATCATCCTCCCCAGCTTCTTCCTCCTCCACGCCTGCATTCCTCGACGAGGCCAACTGGGTCTGTTAGAGTTCAAGCCTATGAATCAATGTCGTCTCCAGATGGTGTTTTACCAGGACAAAGGAAAATTGTCTTTCATGATTGCCCTCATCTTTCCCTTTTCCTCTACAAATTGATTCTTTTTTTCCCACTTAAAGGACAAAAAGCTAACCATAGAATGTTACATATGGGGATGAGAGAAATCCAGTAG

>VvMKK2 (5′-3′)

GTGTTTTTGAAAGTAAAAAAAGAGAGGAGTGAAAGTGGAGGAGGAAAAGTAAACGACGAGGCATGGGTCTTCTTCCTCCTCATTTCTGAAACGCTGACTGGTCTTCTCTTTCACCCACCAAACTTCTTGTATTGGTATTTATAAATTGCAATGCATTAATGTCATAAATCTGAATGTAAATTTATCCAAACCCTAGAGAGGATTGCTTGGTTTCTCAAGACTTTGGAGTTTGGAATCATGAGGAGAGGGCCCTTAAACCCTAGCAATCTCAAGCTCACTCTCCCTCCTGATGAGGATTCATTGACTAAGTTCCTAACTCAGAGTGGGACTTTTATGGATGGTGATCTGCTGGTCAACAGGGATGGAGTTCGAATTGTGTCTAAGAGCGAAGCTGAAGTGCCACCACTTATAAAGCCCTCTGACAACCAGTTGAGCTTGGCTGACATAGACACGATTAAAGTGATTGGGAAGGGCGCCGGTGGAACTGTGCAATTGGTGCAACATAAGTGGACTGGTCAATTTTTTGCGTTGAAGGTTATTCAAATGAATATTCAGGAGGCTGCTCTCAAGCATATTGCACAGGAGCTAAAAATTAACCAGTCATCACAATGCCCATATGTTGTCGTGTGTTACAAGTCTTTCTATGATAATGGTGCCTTTTCTATTATCTTAGAGTACATGGATGGTGGATCTCTTTTAGATTTTCTGAAAAAGGTCAAATCAATTCCAGAGCCGTATCTTGCTGCCATCTGTAATCAGGTGCTCAAGGGTTTGTCGTATCTTCATCATGAAAGACACATCATCCATAGGGACTTAAAACCTTCTAACTTGTTAATAAATCACAGAGGAGAAGTGAAGATCACCGACTTTGGTGTGAGTGCAATACTTACAAGCACCTCTGGGCAGGCAAATACTTTTGTCGGCACGTACAACTATATGTCTCCAGAGAGAATTAGTGGAGGTAAGTATGGCTCCAAAAGTGACATTTGGAGCTTGGGGTTGGTTTTGCTCGAGTGTGCCACTGGTCAGTTCCCGTATTCTCCACCAGAGCAAGGGGAAGGATGGACCAGCTTTTATGAACTTATGGAAGCCATTGTTGACCAGCCACCGCCTTGTGCATCTACTAATCAATTTTCTGCAGAATTCTGCTCTTTCATTTCTGCATGCATACAGAAAGACCCAAATGACAGAAAGTCAGCGCATGAACTTATGGCACATCCTTTTATCAGCATGTACAGAGACTTGAATGTTGATCTTGCAACATACTTCACCAATGCAGGATCTCCACTCGCTACATTTTAAAAGTTTGTCTGAGTTCTTATTTACCAAAAAATAGCATGATCCTAGGAGAAGTGAGGAAATCAGCTATCGGTGTGTCCAGATACCTGGTGAAGGATTCTTAGCGACAATTCTCAAGCCCCTCTCTATGGCAACAACGGCAAGGGGTCTTCAATCATTTGTGACGAACCTCATTGCTTGAAAAATATTGAGTGTGAGAAACATATGAAGAAATTGATGATTTATGACTGCAAGGCAATTAGGCAAATTACTACTGTTTGTATTCTGCCGAAGGCACTTTGGCATGATTTTGAATTGCTCGTTTAGAGGTGCAACACACATCACATTCCCACGAAGAATTTTCCATGTATGTGTAAGATTGTGATAAATACTCAGTTTGAACCCTAAAAAAAAAAAAAAAAAAAAAAAAAAAAAAAA

>VvMKK3 (5′-3′)

TCTCTCTTTCTCTCTCTCACTCGAAAACCCTAGAGAGAAACCCTAATCACAATGAAGAGCAAGAAGCCACTGAAGCAACTGAAGCTCTCTGTTCCTGCTCAAGAAACCCCTATCACCAGTTTCTTGACTGCAAGTGGCACATTTCAGGATGGTGATCTGCTCTTAAACCAGAAGGGTTTGCGGCTTATCTCTGAGGAGAAAGAACCTCGTCCTTCAGAGGCTAAGGAGCTTGATGTACAATTTTCATTGGAAGATCTTGAGACCATCAAAGTCATTGGAAAGGGAAGTGGAGGTGTTGTTCAACTTGTTCGCCACAAATGGGTTGGAACATTATTTGCCTTGAAGGTCATCCAGATGAATATACAAGAGAATTTCCGCAAACAAATTGTGCAGGAGCTGAAGATAAATCAAGCATCACAATGTTCACATATTGTTGTTTGCTACCATTCGTTCTATCACAATGGAGTCATCTCTCTTGTCCTGGAATACATGGATCGTGGATCTTTGGTTGATGTGATCAGACAGGTCAAAACAATCCTTGAACCATATCTTGCTGTTCTTTGTAAGCAGGTTTTACAAGGTCTTGTGTACTTGCACCATGAAAGACACGTAATTCATAGGGACATAAAACCATCCAACCTGCTGGTAAACCACAAAGGGGAAGTAAAGATTACAGATTTTGGTGTGAGTGCTATGCTAGGTAGCTCTATGGGTCAGAGGGACACGTTTGTTGGGACTTACAATTACATGTCGCCTGAGAGAATTAAAGGAAGCACTTATGACTACAGCAGTGATATTTGGAGTCTGGGCATGGTAGCACTTGAATGTGCTATTGGACATTTTCCTTATATGCAATCTGAAGACCAGCAAAGCGGGCCAAGCTTTTATGAGCTCTTAGAGGCAATTGTGGAAAGCCCACCACCATCTGCTCCCCCAGATCAATTCTCTCCAGAATTTTGTTCTTTTATATCTGCCTGCATACAGAAGAACCCTCAAGACCGATTGTCATCATTAGACCTCGTGAGTCACCCTTTCATCAAAAAGTTTGAAGACAAGGACATTGATCTCGAAATTCTGGTAGGCAGCTTGGAACCTCCTGTAAATTTCCCAAGATAGTTTGTCTTGTAATAGTATTCCCAGTGTACCTTTTCCTTCAAAACACGCACTGTTTGTCATCTTGATCAGGTGTTATATGAATTACATCATAGAAGTCAAGCTAGCAAAAAAAAAAAAAAAAAAAAAAAAA

>VvMKK4 (5′-3′)

CTTGAATGCCAAGATCGCAGAAGCAGTGATCCTTGACACACCAAAAATCCCAATTATGGAAACCTAACTGTTACTGGAAAAAAGGACCAGTATAGTAATTAATCATGGCTGGATTAGAGGAATTGAAAAAGAAACTTTCACCATTGTTTGATCCCGATAAAGGCTTATCAGCTGGATCAACTTTGGACCCTTGTGATTCTTATATGCTGTCGGATGGTGGAACAGTTAACTTATTGAGCAAATCATGTGGAGTATACAATATAAATGAGCTCGGGTTGCTAAAGTGCTCATCTTCTCCAGTAGATGAAGCAGATTATAAAGAAAGGACTTATCGGTGTGCTTCCCATGAGATGAGGATTTTTGGAGCCATAGGTAGTGGTGCAAGCAGTGTTGTGCAGAGAGCTATCCATATACCCACTCATAGAATTATTGCGTTGAAGAAGATTAATATTTTTGAAAAGGAGAAAAGGCAACAGCTTCTCACTGAGATAAGGATGCTGTGTGAGGCACCTTGTTATGAGGGCCTTGTGGAGTTTCATGGGGCATTTTATACTCCAGACTCTGGACAGATAAGTATAGCTTTAGAGTACATGGATGGAGGGTCCTTGGCAGATGTCATACGGGTGCAGAAATGTATACCGGAACCAGTTCTTTCATCTATGGTTCGAAAGCTACTACATGGACTATGCTACTTGCATGGAGTTCGACATTTAGTTCATAGAGACATAAAGCCAGCAAATTTGCTAGTAAATCTTAAGGGGGAGCCAAAGATAACAGATTTTGGTATAAGTGCTGGCTTAGAGAATTCTGTGGCAATGTGTGCTACTTTTGTTGGAACTGTTACATATATGTCACCTGAGCGAATTCGGAATGAGAACTATTCTTATCCAGCTGATATTTGGAGCCTTGGTCTTGCACTCTTTGAATGTGGTACAGGGGAATTCCCATATACAGCTAATGAAGGACCTGTTAATCTTATGTTGCAGATACTGGATGATCCATCACCATCTCCACCAAAACACATATTTTCAGCTGAGTTCTGCTCATTTATTGATGCTTGCCTTCAGAAGGATGCAGATGCAAGGCCGACAGCAGAGCAGCTTCTTTCCCACCCATTTATTACAAAGTATGAGCATGCCAGAGTAGATTTAACGGCATTTGTCCGAAGCATTTTTGACCCAACACAAAAGATGAAGGATCTGGCAGATATGCTGATGATACACTATTACTTACTTTTTGATGGGCCTGATGACCTTTGGCAACATACAAAGACCTTATATAAGAAAGATTCAACTTTCAGTTTCTCGGGTAAACAATCTGTTGGTTCAGATGATATTTTTGCTACTTTGTCGGGCATTCGAAGTACATTAGTGGGTGACTGGCCTCCTGAACGACTTGTGCATGTTGTGGAAAAACTTCAGTGCCGTGGTCATGGACAAGATGGAGTTGCCATTCGTGTATCAGGATCCTTTATTGTTGGGAATCAGTTCCTCATATGTGGAGATGGTGTACAAGTAGAAGGTTTGCCTAGTTTTAAAGATCTTTCAATCGAAATTTCTAGCAATCGGATGGGAACATTTCAGGAGCAGTTTATCATGGAACCGGGAGATGCCATTGGCCGCTACTTCATAACCAAACAAGATCTCTACATCATCCAATAGAAAAACTAACAAATAAGATTATGCCCTACAAGGTCAGTAGATTGGGCACTTGTTCACAAGTTTAAAAAAAA

>VvMKK5 (5′-3′)

GTTATCAACTATAGCCTCACCCATCGGCGCAAGTTAGCACATGGCAGTAGTCCGAGACCGCCGGCAGCTCAACCTCCGCCTCCCCTTGCCTGAAATCTCCGAGCGCCGTCCCCGATTCCCACTTCCCCTTCCTCCCACCAATCTCCCGTTCTCCTCCGGCGCCACTACCGCCGCCGACCTTGAGAAGATCGAAGTGATCGGCCACGGGAACGGCGGCACCGTCTACAAGGTCCAGCACAAGCGCACTGCAGCGAACTACGCGCTCAAGGTGGTCCACGGCGACTGCGACCCCACCGTCCGCCGTCAGGTGTTGCGGGAGATGGAGATCCTCCGATTTACGGACTCGCCGTTTGTGGTCCAGTGTCACGGCATCTTCCAGAAGCCCTCCGGTGACATCGCGATTTTAATGGAGTACATGGACGCCGGTACCTTAAAAACCCTCCTGGAAACCAAAGGCACTTTCTCGGAAGTGGATCTCGCTGGCGTCGCCGGTCAGGTGCTGAACGGCCTCAGTTACCTCCACAGCCACAAGATCATCCACAGAGACATCAAACCCGCAAATCTTTTGGTGAACGGGAACATGGAGGTGAAGATCGCCGATTTCGGAGTGAGCAAGATCATGCGCCGCATGCTCGACTCTTGCAACTCCTACGTGGGCACCTGCGCCTACATGAGCCCGGAGCGGTTCGACCCCGACTCCCACGGCTCCAATTACGACGGCTACTCCGGCGATATCTGGAGCTTAGGTCTCACCCTATTAGAACTCTACGTCGGTCACTTTCCCTTACTGCCGGCCGGACAGAAGCCGGACTGGGCAACACTCATGTGCGCCATATGCTTCGGCGAGGAGCCGGCGCTGCCAGAGGGCGTATCGGAGGAGTTCCGGAACTTCATCGAGTGTTGTCTACAGAAGGATTCAACAAAGAGGTGGACGGCGGCTCAGCTTCTATCGCATCCGTTCATCTGTAAACAATCTGTCATCTGAGCCGATGATCTGATCTGACCTTCAAAAAACGAGATCGATGATCTGGAGTTGTGTATATACCAATCGGAATCAGAATCAGAATCTTCTGTATTTTTAGTTGTTTGTACATTGTCTTAAGCTCCTTGTTCACAGTAAATCAGATGCGAAAAGAGAAGCTTAGATTCAATTAAATCTTCCTTTTATC
