# Supplementary figures and images for: Cloning, molecular and functional characterization by overexpression in Arabidopsis of MAPKK genes from grapevine (Vitis vinifera)
Source: BMC Plant Biol. 2020 May 7;20:194. doi: 10.1186/s12870-020-02378-4 (PMC7203792; doi:10.1186/s12870-020-02378-4)

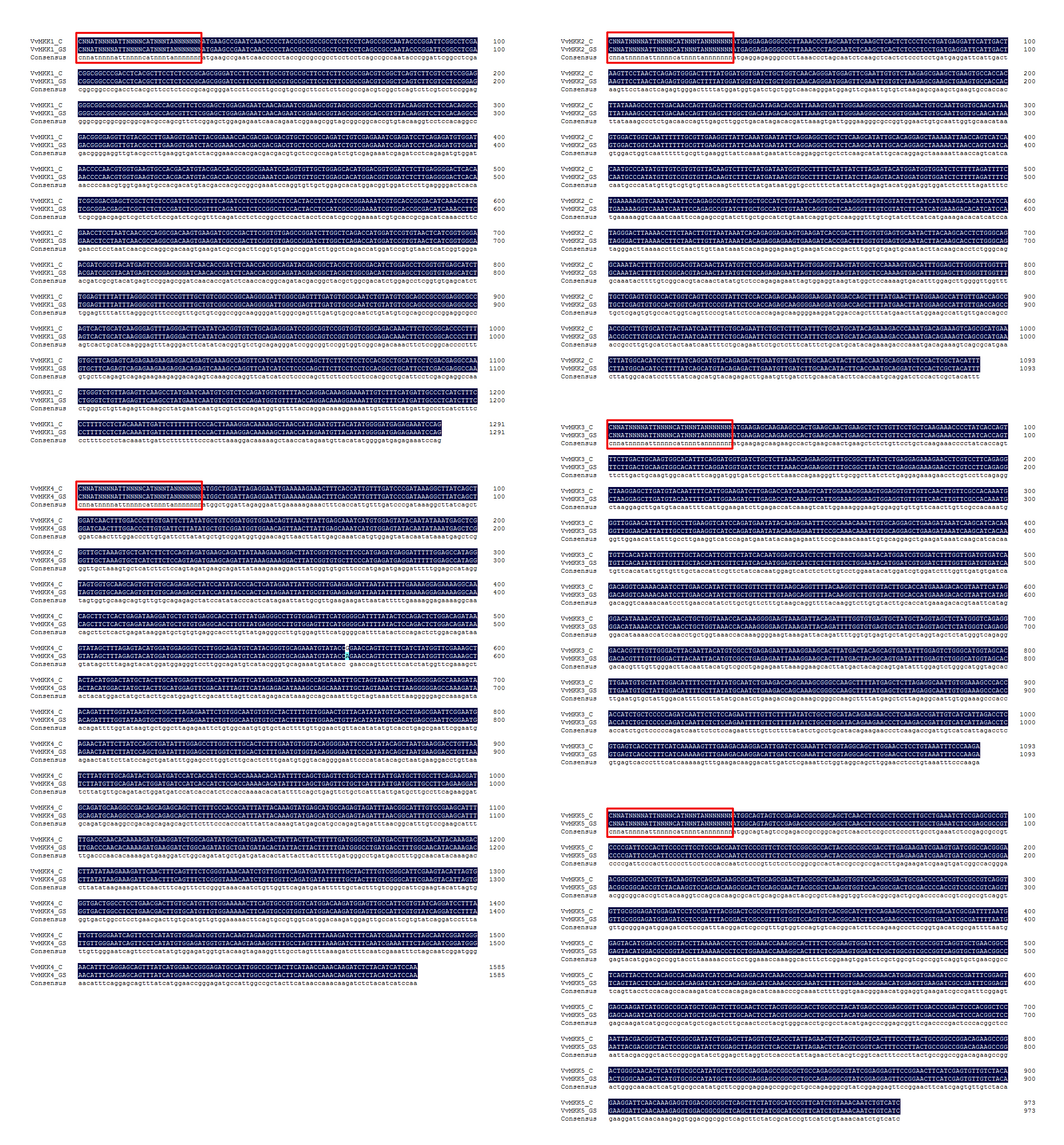

Supplement: Supplementary file 2 — Additional files 2 : Figure S1. Alignment of nucleic sequences of each of the cloned VvMKK genes and the corresponding sequence of the predicted gene in the grapevine whole genome sequence database. Sequence homologies are highlighted in black while differences are highlighted in grey. [file 12870_2020_2378_MOESM2_ESM.jpg]

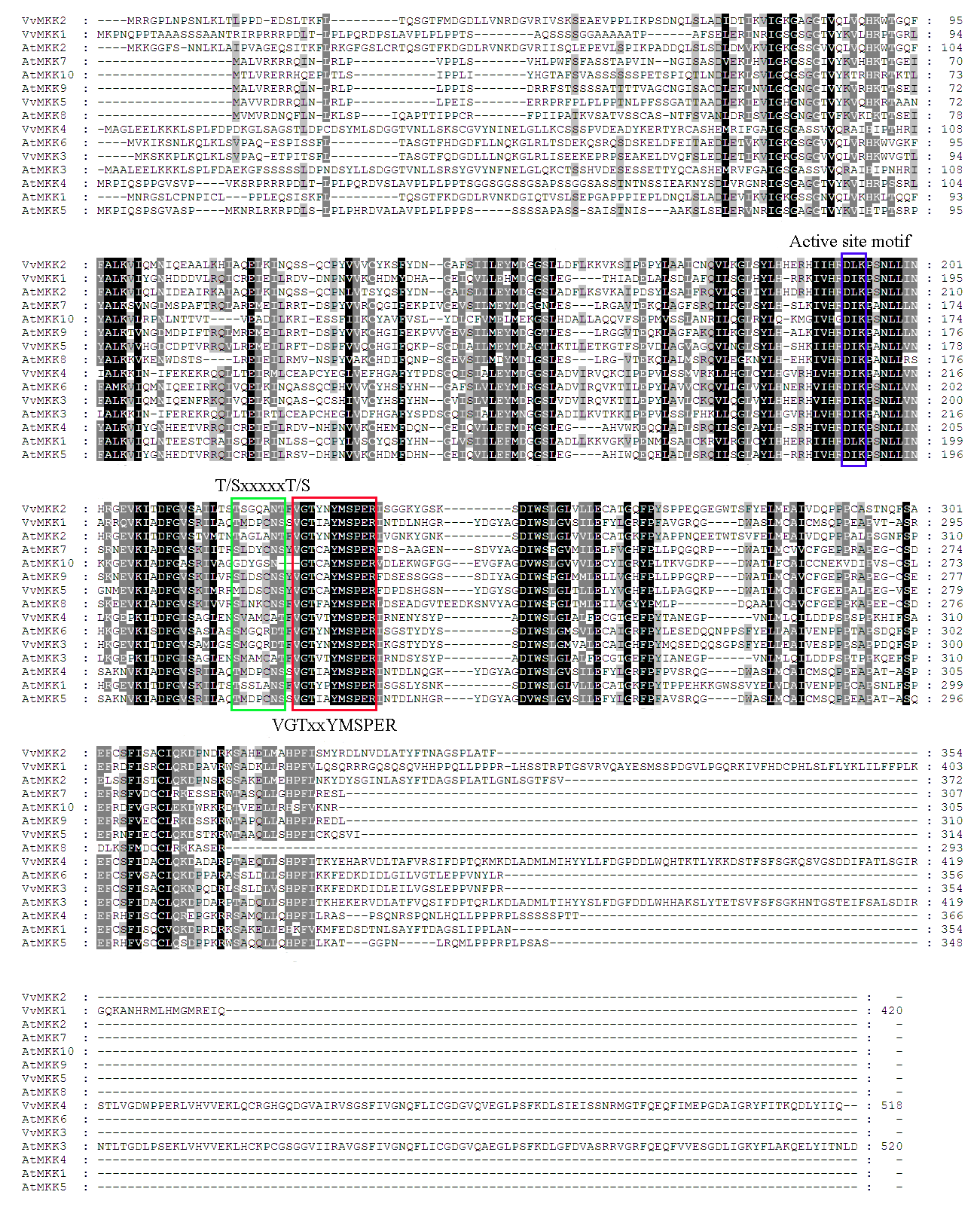

Supplement: Supplementary file 3 — Additional files 3 : Figure S2. Alignment of amino acid sequences and domain analysis of MAPKK genes from grapevine and Arabidopsis. Alignment was performed using ClustalW program. The red-box indicates the conserved signature motif, the green-box indicates the plant MAPKK specific motif -T/SXXXXXS/T- and the active site -D (I/L/V) K- motif is highlighted in blue. [file 12870_2020_2378_MOESM3_ESM.tif]

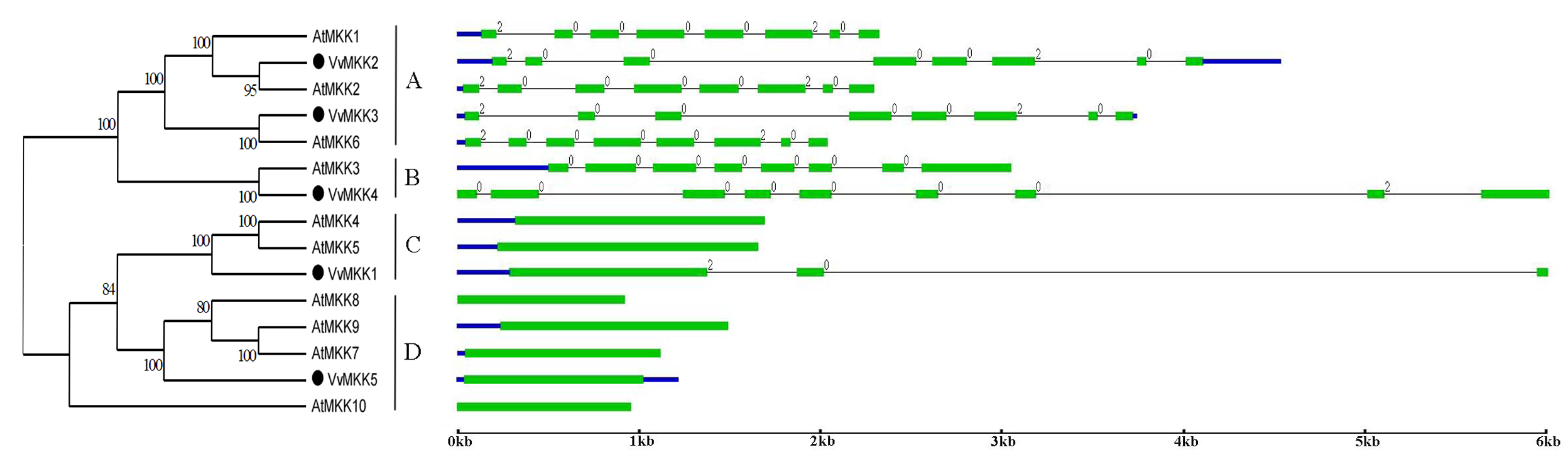

Supplement: Supplementary file 4 — Additional files 4 : Figure S3. Phylogenetic relationship and schematic diagram of intron/exon structure of MAPKK genes in grapevine and Arabidopsis. The phylogenetic tree (left panel) was created using MEGA5.0 software with the neighbor-joining (NJ) method. Bootstrap values for 1000 replicates are indicated at each branch. Letters A-D on the right indicate different groups of MAPKKs. Exon/intron structures of the MAPKK genes are shown in the right panel. The green boxes indicate exons, while the single lines indicate introns. UTRs are indicated by thick blue lines at both ends. 0, 1 and 2 represented different intron phases. Gene models are drawn to scale as indicated on the bottom of the figure. [file 12870_2020_2378_MOESM4_ESM.tif]
