# Supplementary material for: Cloning, molecular and functional characterization by overexpression in Arabidopsis of MAPKK genes from grapevine (Vitis vinifera)
Source: BMC Plant Biol. 2020 May 7;20:194. doi: 10.1186/s12870-020-02378-4 (PMC7203792; doi:10.1186/s12870-020-02378-4)
Supplement: Supplementary file 5 — Additional files 5 : Table S2. The number of MAPKK gene family members in different plant species. [file 12870_2020_2378_MOESM5_ESM.docx]

Table S2. The number of the MAPKK gene family in different species

| Species | Group A | Group B | Group C | Group D | Total |
| --- | --- | --- | --- | --- | --- |
| *Arabidopsis* | 3 | 1 | 2 | 4 | 10 |
| Apple | 3 | 1 | 2 | 3 | 9 |
| Banana | 1 | 3 | 3 | 3 | 10 |
| *Brassica rapa* | 5 | 1 | 4 | 1 | 11 |
| *B. distachyon* | 2 | 3 | 2 | 5 | 12 |
| Canola | 3 | 1 | 2 | 1 | 7 |
| Chinese jujube | 2 | 1 | 0 | 2 | 5 |
| Cotton | 4 | 1 | 2 | 4 | 11 |
| Cucumber | 3 | 1 | 1 | 1 | 6 |
| Grapevine | 2 | 1 | 1 | 1 | 5 |
| Maize | 2 | 3 | 2 | 2 | 9 |
| Poplar | 3 | 1 | 2 | 5 | 11 |
| Rice | 2 | 1 | 2 | 3 | 8 |
| Tomato | 2 | 1 | 1 | 1 | 5 |
| Watermelon | 3 | 1 | 1 | 1 | 6 |
