# Supplementary material for: Cloning, molecular and functional characterization by overexpression in Arabidopsis of MAPKK genes from grapevine (Vitis vinifera)
Source: BMC Plant Biol. 2020 May 7;20:194. doi: 10.1186/s12870-020-02378-4 (PMC7203792; doi:10.1186/s12870-020-02378-4)
Supplement: Supplementary file 6 — Additional files 6 : Table S3. The primer sequences used to clone the MAPKK genes in grapevine by PCR. [file 12870_2020_2378_MOESM6_ESM.docx]

Table S3. The primer sequences used to clone the MAPKK genes in grapevine by PCR

| Name | Forward primers (5′ - 3′) | Reverse primers (5′ - 3′) |
| --- | --- | --- |
| VvMAPKK1 | CGTACCCATGAAACCACAGC | CTACTGGATTTCTCTCATCCCCAT |
| VvMAPKK2 | ATGAAGAGCAAGAAGCCACTG | CTATCTTGGGAAATTTACAGGAGG |
| VvMAPKK3 | ATCATGGCTGGATTAGAGG | TGAACAAGTGCCCAATCTAC |
| VvMAPKK4 | GAGTTTGGAATCATGAGGAG | TTAAAATGTAGCGAGTGGAG |
| VvMAPKK5 | AGCACATGGCAGTAGTCCGAG | TCAGATGACAGATTGTTTACAGATG |
