# Supplementary material for: Cloning, molecular and functional characterization by overexpression in Arabidopsis of MAPKK genes from grapevine (Vitis vinifera)
Source: BMC Plant Biol. 2020 May 7;20:194. doi: 10.1186/s12870-020-02378-4 (PMC7203792; doi:10.1186/s12870-020-02378-4)
Supplement: Supplementary file 7 — Additional files 7 : Table S4. The primer sequences used for 3′ RACE of the MAPKK genes in grapevine. [file 12870_2020_2378_MOESM7_ESM.docx]

Table S4. The primer sequences of the MAPKK genes in grapevine for 3′ RACE

| **Name** | **Forward primers (5′ - 3′)** | **Reverse primers (5′ - 3′)** |
| --- | --- | --- |
| VvMAPKK1 | TCAACCACGGCAGATACGACGGCTAC | TTAGGGCGTTTCCCGTTTGCTGT |
| VvMAPKK2 | CAGCAGTGATATTTGGAGTCTGGGCAT | GCAATCTGAA GACCAGCAAAGCGGG |
| VvMAPKK3 | GCAGAGCAGCTTCTTTCCCACCCATT | ATCGGATGGGAACATTTCAGGAGCAG |
| VvMAPKK4 | CAGCCACCGCCTTGTGCATCTACTAATC | TGCCACTGGTCAGTTCCCGTATTCTCC |
| VvMAPKK5 | GGTCCAGTGTCACGGCATCTTCC | GCCTCAGTTACCTCCACAGCCAC |
| 3′ RACE | GGTGGTAGAGCTCGCAGGACTGCAGCTGACTG | AGAGCTCGCAGGACTGCAGCTGACTGACTAC |
