# Supplementary material for: Cloning, molecular and functional characterization by overexpression in Arabidopsis of MAPKK genes from grapevine (Vitis vinifera)
Source: BMC Plant Biol. 2020 May 7;20:194. doi: 10.1186/s12870-020-02378-4 (PMC7203792; doi:10.1186/s12870-020-02378-4)
Supplement: Supplementary file 8 — Additional files 8 : Table S5. The primer sequences of the MAPKK genes in grapevine for quantitative RT-PCR. [file 12870_2020_2378_MOESM8_ESM.docx]

Table S5. The Primer sequences of the MAPKK genes in grapevine for quantitative RT-PCR

| **Name** | **Forward primers (5′ - 3′)** | **Reverse primers (5′ - 3′)** |
| --- | --- | --- |
| VvMKK1 | GGGATTGGGCGAGTTTGATGTGC | CAGGCGTGGAGGAGGAAGAAGC |
| VvMKK2 | TACAGAAGAACCCTCAAGACCG | TCAAGATGACAAACAGTGCGTG |
| VvMKK3 | GGAACATTTCAGGAGCAGTTTATC | TGTGAACAAGTGCCCAATCTAC |
| VvMKK4 | TTATGGCACATCCTTTTATCAGC | GAAGACCCCTTGCCGTTGTT |
| VvMKK5 | CAGAGGGCGTATCGGAGGAGTTC | AGGTCAGATCAGATCATCGGCTCAG |
| Actin | TACAATTCCATCATGAAGTGTGATG | TTAGAAGCACTTCCTGTGAACAATG |
