# Supplementary material for: Cloning, molecular and functional characterization by overexpression in Arabidopsis of MAPKK genes from grapevine (Vitis vinifera)
Source: BMC Plant Biol. 2020 May 7;20:194. doi: 10.1186/s12870-020-02378-4 (PMC7203792; doi:10.1186/s12870-020-02378-4)
Supplement: Supplementary file 9 — Additional files 9 : Table S6. The primer sequences used to insert VvMKK2 and VvMKK4 genes into transformation vectors. [file 12870_2020_2378_MOESM9_ESM.docx]

Table S6. The Primer sequences for cloning of VvMKK2 and VvMKK4 genes in vectors

| Genes | Forward（5′-3′） | Reverse（5′-3′） |
| --- | --- | --- |
| VvMKK2 | CGGGATCCCGATGAGGAGAGG | CGAGCTCGTTAAAATGTAGCGAGTGG |
|  | (*BamH*I site is underlined) | (*Sal*I site is underlined) |
| VvMKK4 | CGGGATCCCGATGGCTGGATTAGAG | CGAGCTCGCTATTGGATGATGTAGAG |
|  | (*BamH*I site is underlined) | (*Sal*I site is underlined) |
